# Assessing the diagnostic accuracy of unilateral systematic biopsy combined with targeted biopsy

**DOI:** 10.3389/fonc.2025.1599869

**Published:** 2025-08-19

**Authors:** Qiyou Wu, Chunlei He, Xiang Tu, Bo Chen, Jinjiang Jiang, Jinbao Wang, Zhouhaoran Chen, Ruoxuan Liu, Qiaoxue Huang, Bo Tang, Jin Yao, Qiang Wei

**Affiliations:** ^1^ Department of Urology, Institute of Urology, West China Hospital, Sichuan University, Chengdu, Sichuan, China; ^2^ Department of Radiology, West China Hospital, Sichuan University, Chengdu, Sichuan, China; ^3^ West China School of Medicine, Sichuan University, Chengdu, Sichuan, China; ^4^ Department of Nursing, Xiufeng Community Health Service Center, Guilin, Guangxi, China

**Keywords:** prostate cancer, prostate biopsy, multiparametric magnetic resonance imaging utilization, unilateral index lesion, regional biopsy

## Abstract

**Background:**

To evaluate unilateral systematic biopsy (SB) combined with targeted biopsy (TB) and assess its diagnostic accuracy in a real-world, single-centre setting.

**Methods:**

Patients with ≥1 MRI lesion who underwent both transperineal 12-core and 3-core TB were enrolled in this study. Detection rates for total prostate cancer (PCa) and clinically significant PCa (csPCa) were compared between TB, unilateral SB+TB, and SB+TB. Pathological consistency was assessed using the kappa test, and logistic regression was used to identify potential predictors.

**Results:**

A total of 250 men were enrolled, of which 126 (50.4%) and 103 (41.2%) exhibited total PCa and csPCa, respectively. Compared to SB+TB, ipsilateral SB combined with TB (ips-SB+TB) had a comparable csPCa detection rate (99/250 vs 103/250, p=0.125), while fewer clinically insignificant PCa were detected (17/250 vs 23/250, p=0.031). In addition, ips-SB+TB demonstrated superior sensitivity for csPCa (96.1%) with an AUC of 0.98. The ips-SB+TB had a significantly higher positive core rate than SB+TB (472/2244 vs. 563/3744, p<0.001). Moreover, ips-SB+TB also had a high consistency of Gleason grade compared to SB+TB (Kappa=0.89). In the multi-lesion cohort, ips-SB+TB also had a comparable csPCa detection rate compared to SB+TB (63/128 vs. 67/128, p=0.125).

**Conclusions:**

In conclusion, our study showed that ips-SB+TB was comparable to SB+TB in detecting csPCa. The results of this study provide valuable insight into the potential of ips-SB+TB as an alternative to SB+TB.

## 1 Introduction

Prostate cancer (PCa) is one of the most common malignancies and the second leading cause of cancer-related deaths among men (1). However, the diagnosis of PCa requires complete biopsy of the entire organ, rather than just the lesion identified on imaging, because of the overlap between the appearance of benign and malignant lesions on imaging, leading to an increased risk of overtreatment (2). The utilization of multiparametric magnetic resonance imaging (mpMRI) and the adoption of the Prostate Imaging Reporting and Data System version 2.1 (PI-RADS v2.1) criteria have transformed the diagnosis of PCa, aiding in the detection and localization of PCa and significantly improving the detection rate of clinically significant PCa (csPCa) (3–5). To date, MRI/ultrasound (US) fusion targeted biopsy (TB) is determined to increase the detection rates of csPCa while decreasing the detection rates of clinically insignificant PCa (ciPCa) (4–6). The combination of systematic prostate biopsy (SB) and TB (SB+TB) leads to a higher detection rate of PCa, demonstrating the significant added value of both biopsy methods (5–7).

In accordance with the European Association of Urology (EAU) guidelines for 2024, the regional biopsy (RB) concept, which involves performing TB with perilesional sampling in patients with PI-RADS≥4, was recommended. SB+TB which was previously recommended, has been shown to enhance the detection rates of PCa. However, this approach also results in increased biopsy expenses, the detection of clinically insignificant PCa (ciPCa), and subsequent overtreatment among patients with low-grade PCa (3, 8, 9).

Several recent studies have investigated the potential of regional biopsy (RBx) combined with TB regimens, including ipsilateral SB (ips-SB) combined with TB (ips-SB+TB), contralateral SB (con-SB) combined with TB (con-SB+TB), saturation TB as substitutes for SB+TB. These studies have revealed that ips-SB+TB and saturation TB with fewer cores increases detection rates of csPCa while decreasing detection rates of ciPCa (8, 10–19). Few studies have suggested that distant systematic biopsy cores have limited impact in detecting csPCa and the biopsy should only be performed on the same side as the target lesion identified on MRI. However, most of these studies focused on single lesions and failed to use the established PI-RADS v2.1 scoring system. The objective of this study is to evaluate the detection rate of csPCa using different prostate sampling methods and to confirm whether ips-SB+TB could yield similar csPCa detection rates as SB+TB among patients with unilateral mpMRI-visible lesions.

## 2 Methods

### 2.1 Patient selection

We retrospectively reviewed the patients who were prospectively enrolled for prostate biopsy at our institution from September 2022 to June 2023, and also included our prostate biopsy database (2018-2022). We enrolled patients who had prebiopsy MRI visible targets. The patients who met the following inclusion criteria were included: (1) with unilateral index lesion; (2) having a PI-RADS v2.1 score≥3 and (3) clinical stage<T3. The exclusion criteria were as follows: (1) Patients who performed MRI scans at other institutions; (2) those with poor-quality MRI scans to identify prostate; (3) patients who had received prior relevant treatments including surgery, drug therapy, or radiotherapy before the prostate biopsy (**Supplementary Figure 1**). Baseline clinical and demographic variables included age, biopsy setting, free PSA (fPSA), total PSA, PSA density (PSAD), prostate volume (PV) (calculated by ellipsoid formula), maximum cancer core length, frequency of prostate biopsy, highest PI-RADS lesion score, number of PI-RADS lesions. Patient recruitment and informed consent were obtained from all patients in our hospital prior to the biopsy procedure.

### 2.2 Standard of reference and main outcome

SB+TB was defined as the standard reference of the biopsy and its pathology result was defined as the final standard reference. The main outcomes were detection rate of PCa and sensitivity for TB, SB, TB+SB, ips-SB+TB and con-SB+TB to detect PCa. Additionally, the consistency of the pathology was also assessed.

### 2.3 Prebiopsy magnetic resonance imaging and analysis

Before biopsy, we conducted a 3.0T mpMRI following our institution’s protocol, which has been previously reported in detail. The highest b-value of diffusion weighted imaging was 1,400 s/mm^2^. Two radiologists, with 5 and 7 years of experience respectively on genitourinary imaging, blindly reviewed all MRI scans according to the PI-RADS v2.1 criteria. When discrepancies occurred, they consulted a senior radiologist (J. Yao) to reach a consensus (20, 21).

### 2.4 Transperineal prostate biopsy

Prostate biopsy, which included transperineal TB and SB under local anaesthesia of a periprostatic nerve block of 20 cc 1% lidocaine using a 16-gauge needle (Bard biopsy system). The sonographer who performed the TRUS-guided procedure had more than 5 years of experience in genitourinary medicine. Before the biopsy, the urologist and radiologist re-evaluated all MRIs to match the index lesion, which is defined as the lesion with the highest PIRADS v2.1 score or the largest lesion volume among all lesions with the same PIRADS v2.1 score. Firstly, a 3-core MRI/US fusion TB is performed for the index lesion, followed by a 12-core SB. The protocol for SB has been described in detail elsewhere. Briefly, 5 cores are taken from each side of the peripheral zone and 1 core from each side of the transitional zone, resulting in a total of 12 cores bilaterally. The unilateral SB is defined using the unilateral 6 cores of the SB (**Supplementary Figure 2**).

Each biopsy sample was collected in a separate container and evaluated by a group of genitourinary pathologists with over 10 years of experience. The Gleason score (GS) of each positive core was labelled. csPCa was defined as GS score 3 + 4 or higher (22).

### 2.5 Statistical analysis

Frequencies and median (interquartile range, IQR) or mean ± standard deviation (SD) were used to express categorical and continuous variables, respectively. The detection and the sensitivity of overall PCa and csPCa by 12-core SB, 3-core TB, and unilateral SB + 3-core TB were compared for the overall cohort using the McNemar paired test. Pathology consistency categorized by Gleason grade group (GG) was evaluated using the Kappa test. The binary logistic regression with Forward LR method was performed to identify predictors of the csPCa missed by ips-SB+TB.

All analyses were two-sided, with statistical significance set at p<0.05. Statistical analyses were performed using IBM SPSS Statistics version. The statistical analysis was performed using R version 4.3.2 (R Foundation for Statistical Computing, Vienna, Austria).

## 3 Results

### 3.1 Baseline characteristics of patients

A total of 250 patients were final enrolled in this study. 85.6% of the patients (214/250) were biopsy-naïve. The median prebiopsy total PSA was 9.65 (IQR: 6.99~14.00) ng/ml. 122 (48.8%) patients exhibited single lesion on MRI, while the remaining 118 (47.2%) exhibited 2 lesions and 10 (4.0%) exhibited 3 lesions. Demographic and clinical characteristics of all patients are shown in **Table 1**.

**Table 1:** Clinical and demographic information.

Characteristics	Total (n=250)	No PCa (n=124)	PCa (n=126)	P value
Mean ± SD age, Years	66.02 ± 8.53	65.39 ± 8.43	66.63 ± 8.62	0.25
Mean ± SD Height	1.67 ± 0.06	1.67 ± 0.06 (98/124)	1.67 ± 0.06 (118/126)	0.91
Weight	66.34 ± 8.09	67.06 ± 8.17	65.74 ± 8.01	0.23
BMI	23.77 ± 2.52	23.99 ± 2.45	23.59 ± 2.57	0.27
Median prebiopsy free PSA(IQR), ng/ml	1.41 (0.88-1.99)	1.48 (0.98-2.06)	1.32 (0.83-1.94)	0.038
Median prebiopsy total PSA (IQR)	9.65 (7.00-14.02)	8.88 (6.42-12.5)	10.30 (7.29-14.3)	0.041
Median PSA density (IQR), ng/ml/cc	0.19 (0.125-0.34)	0.14 (0.11-0.20)	0.28 (0.19-0.44)	<0.001
Median ml prostate volume (IQR)	48.14 (32.21-70.48)	62.65 (48.22-81.96)	35.85 (27.43-48.08)	<0.001
Median maximum cancer core length (IQR), cm	1.40 (1.00-1.80)	1.40 (1.00-1.80)	1.40 (1.02-1.80)	0.86
Frequency of prostate biopsy				0.01
1	214	99	115	
2	33	22	11	
3	3	3	0	
PI-RADS score distribution on mpMRI, n (%)				<0.001
PI-RADS 3 lesions	76	62	14	
PI-RADS 4 lesions	106	47	59	
PI-RADS 5 lesions	68	15	53	
Number of targets on MRI, n (%)				<0.001
1	122	78	44	
2	118	41	77	
3	10	5	5	

median (Q1-Q3).

### 3.2 Detection rate and sensitivity of PCa

**Figure 1:**
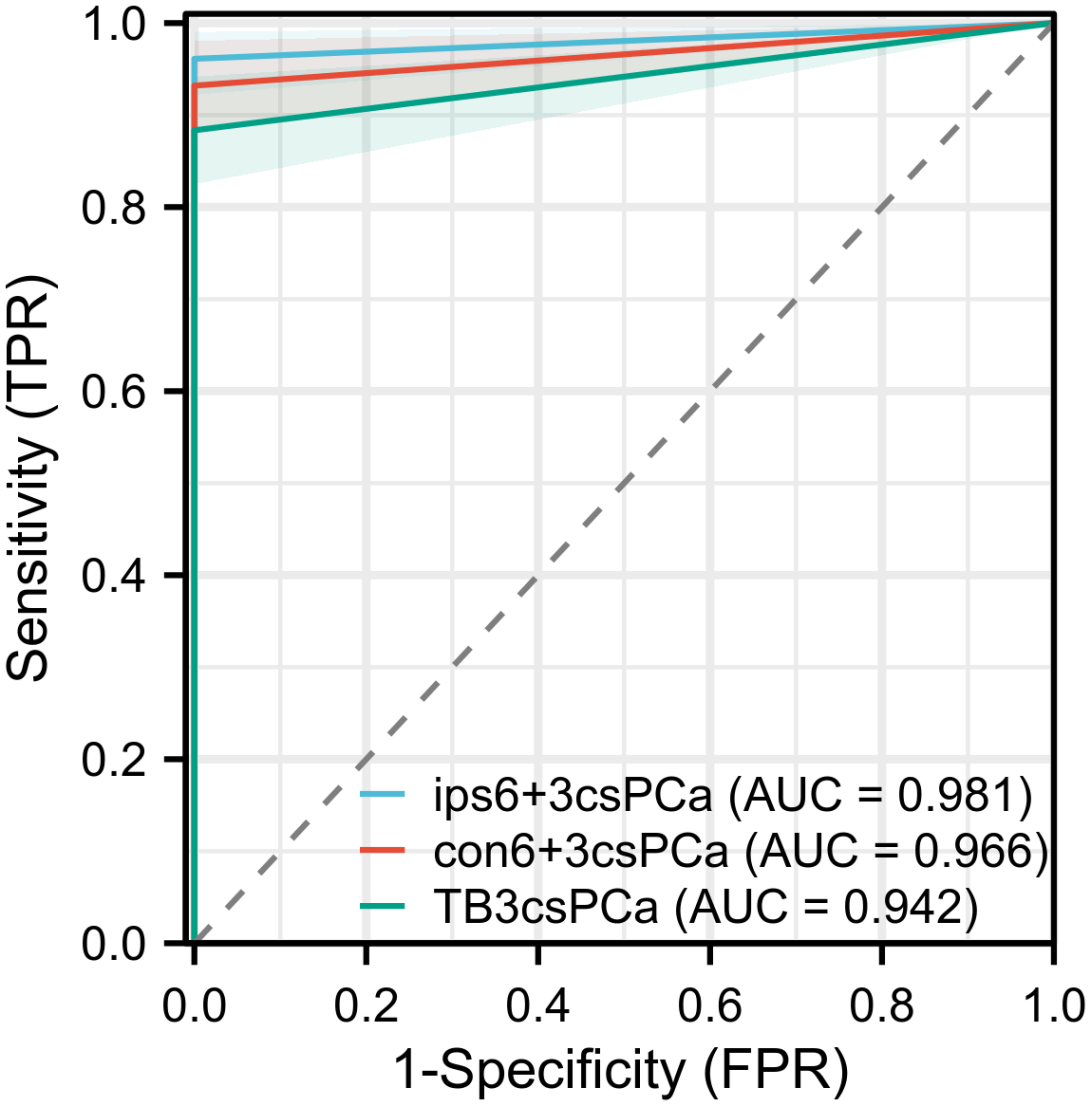
The ROC curve of the diagnostic performance of different biopsy methods for detecting csPCa.

Among the 250 patients, PCa was detected in 126 (50.4%) using SB+TB. Of these, csPCa and ciPCa were detected in 103 (41.2%) and 23 (9.2%) patients respectively. Compared to SB+TB, the ips-SB+TB approach detected significantly fewer cases of PCa (46.4%, p = 0.002) and ciPCa (6.8%, p = 0.031), while the detection rate for csPCa remained comparable (39.6%, p = 0.125). 4 patients with csCPa were missed by ips-SB+TB and their characteristics were displayed in **Supplementary Table 1**. Conversely, SB+TB demonstrated a higher detection rate for csPCa than TB (36.4%, p<0.001) and con-SB+TB (38.4%, p = 0.016) (**Table 2**). Additionally, ips-SB+TB exhibited a superior sensitivity for detecting csPCa (96.1%) with an AUC of 0.981, when compared to con-SB+TB and TB. (**Figure 1**).

**Table 2:** Detection rate and sensitivity of PCa for different biopsy schemes.

Biopsy strategies	Detection rate n (%)	P value		Sensitivity n (%)	95% CI	P value	
		Compared with SB+TB	Compared with TB			Compared with ips-SB+TB	Compared with TB
Any PCa (ISUP GG ≥ 1)							
SB+TB	126 (50.4)	-	< 0.001	126	-	-	-
TB	105 (42.0)	< 0.001	–	105 (83.3)	0.76-0.89	< 0.001	–
ips-SB+TB	116 (46.4)	0.002	< 0.001	116 (92.1)	0.86-0.96	-	< 0.001
con-SB+TB	121 (48.4)	0.063	< 0.001	121 (96.0)	0.91-0.99	0.302	< 0.001
ips-SB	99 (39.6)	< 0.001	0.345	99 (78.6)	0.70-0.85	< 0.001	0.345
con-SB	70 (28.0)	< 0.001	< 0.001	70 (55.6)	0.46-0.64	< 0.001	< 0.001
csPCa (ISUP GG ≥ 2)							
SB+TB	103 (41.2)	–	< 0.001	103	–	–	–
TB	91 (36.4)	< 0.001	-	91 (88.3)	0.81-0.94	0.008	-
ips-SB+TB	99 (39.6)	0.125	0.008	99 (96.1)	0.90-0.99	–	0.008
con-SB+TB	96 (38.4)	0.016	0.063	96 (93.2)	0.87-0.97	0.549	0.063
ips-SB	80 (32.0)	< 0.001	0.054	80 (77.7)	0.68-0.85	< 0.001	0.543
con-SB	46 (18.4)	< 0.001	< 0.001	46 (44.7)	0.39-0.55	< 0.001	< 0.001
ciPCa (ISUP GG 1)							
SB+TB	23 (9.2)	-	< 0.001	23	-	-	-
TB	14 (5.6)	< 0.001	–	8 (34.8)	0.16-0.57	0.031	–
ips-SB+TB	17 (6.8)	0.031	0.063	14 (60.9)	0.39-0.80	-	0.031
con-SB+TB	25 (10.0)	1	0.002	21 (91.3)	0.72-0.99	0.065	< 0.001
ips-SB	19 (5.2)	0.523	0.405	10 (43.5)	0.23-0.66	0.125	0.754
con-SB	24 (9.6)	1	0.100	14 (60.9)	0.39-0.80	1	0.238

### 3.3 Positive cores rate and pathology consistency

**Figure 2:**
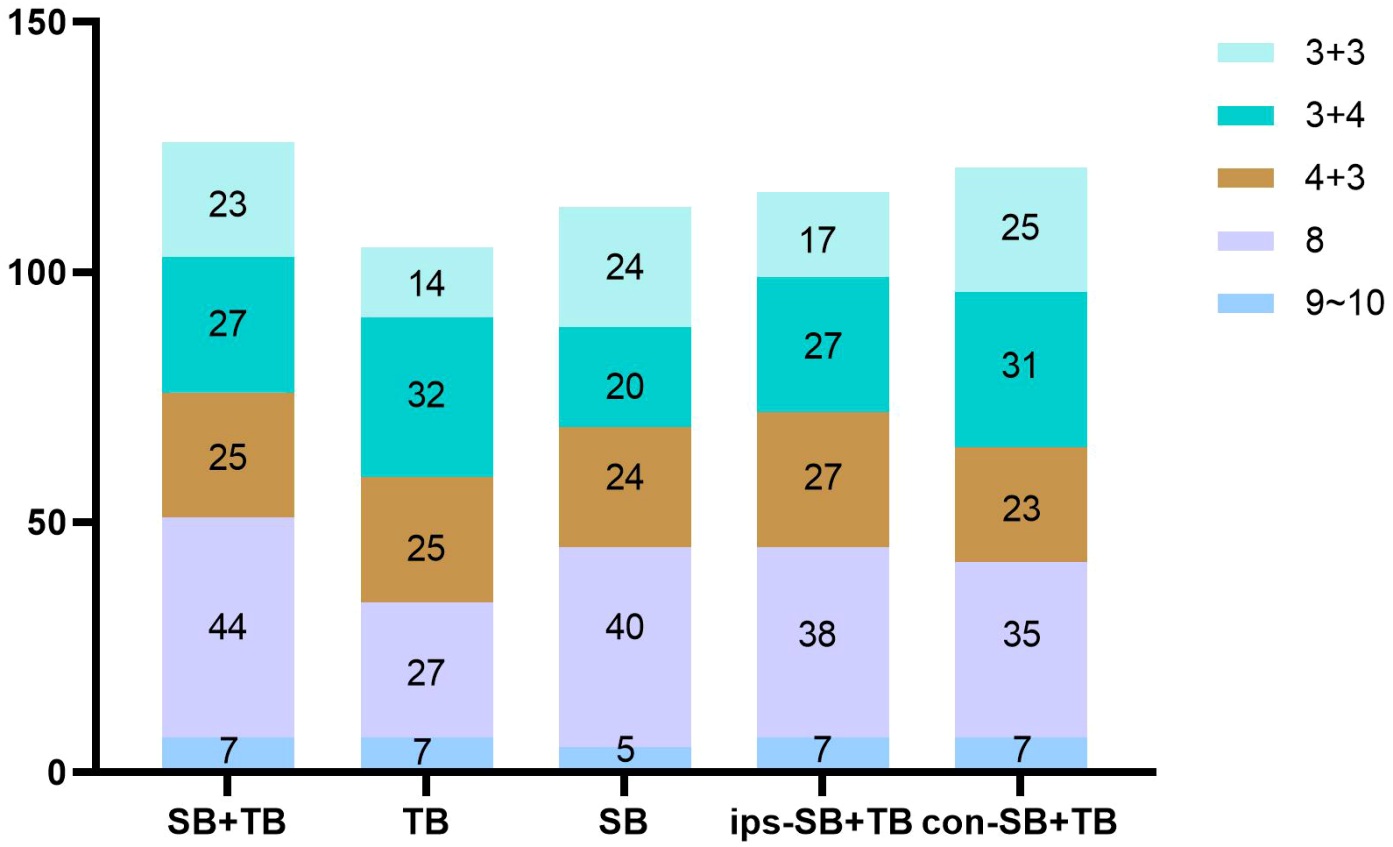
The distribution of gleason scores of PCa detected by different biopsy methods.

The ips-SB+TB (21.0%, 472/2244) exhibited a significant higher positive cores rate than SB+TB (15.0%, 563/3744) and con SB+TB (13.9%, 312/2244) (p<0.001) (**Supplementary Table 2**). The distribution of the GS for each biopsy method could be seen in **Figure 2**. Compared with SB+TB, ips-SB+TB underestimated 18 cases of PCa and missed 4 cases of csPCa, while con-SB+TB underestimated 26 cases of PCa and missed 24 cases of csPCa. (**Table 3**) Additionally, ips-SB+TB also demonstrated highest consistency of GG compared with SB+TB (Kappa=0.89, 95% CI: 0.85, 0.92), con-SB+TB (Kappa=0.85, 95% CI: 0.82, 0.89) and TB (Kappa=0.70, 95% CI: 0.66, 0.74), which had relatively lower consistency of GG. (**Supplementary Table 3**)

**Table 3:** Cross-tabulation of pathology grades between ips-SB+TB and SB+TB, and con-SB+TB and SB+TB.

		Ips-SB+TB					
	ISUP	0	1	2	3	4	5
SB+TB	**0**	124	0	0	0	0	0
	**1**	9	14	0	0	0	0
	**2**	0	2	25	0	0	0
	**3**	1	0	1	23	0	0
	**4**	0	1	1	3	39	0
	**5**	0	0	0	0	0	7
		con-SB+TB					
	ISUP	0	1	2	3	4	5
SB+TB	**0**	124	0	0	0	0	0
	**1**	2	21	0	0	0	0
	**2**	1	4	22	0	0	0
	**3**	1	0	8	16	0	0
	**4**	1	0	1	8	34	0
	**5**	0	0	0	0	0	7

### 3.4 Subgroup analysis

Subgroup analysis was performed based on PI-RADS score, PSA level, PSAD level, biopsy history and the number of lesions. The results revealed that only ips-SB+TB had a comparable csPCa detection rate than SB+TB, not con-SB+TB and TB. (**Supplementary Table 4**). Additionally, we assessed the influence of lesion number on the detection rate of ips-SB+TB, and found that patients with multiple lesions were more likely to have csPCa than those with a single lesion (63/128 vs 36/122, p=0.002), especially in the patient with a PI-RADS 5 lesion (**Supplementary Figure 3**).

### 3.5 Potential predictors of PCa underestimated by ips-SB+TB

The potential predictors of csPCa underestimated by ips-SB+TB (fPSA, tPSA, PSAD, prostate volume, biopsy history, PI-RADS score, lesion size and number of leison) were evaluated. However, no significant predictors were found in multivariable analysis. (**Supplementary Table 5**)

## 4 Discussion

Our study indicated that ips-SB+TB had a comparable detection rate for csPCa than SB+TB, while simultaneously reducing the detection of ciPCa and unnecessary cores. Additionally, ips-SB+TB demonstrated a higher positive cores rate for csPCa, which is consistent with the previous studies (8, 10, 11, 13, 15, 23). On the contrary, a significant number of csPCa were missed by con-SB+TB, resulting in relatively more ciPCa being diagnosed.

Moreover, our study also revealed that the ips-SB+TB had ideal detection efficiency for patients with multi-lesions, which has not been reported in the previous studies. In subgroup analyses, our results showed that the csPCa detection rate of ips-SB+TB was comparable than that of SB+TB in patients with both single-and multi-lesions.

Therefore, our study suggests that ips-SB+TB may be a suitable alternative to SB+TB for patients with unilateral index lesion, avoiding unnecessary biopsy cores and overdiagnosis of ciPCa. Furthermore, our study supports previous research indicating that relying solely on TB would miss a considerable number of patients with csPCa, SB should not be omitted due to its additional value in detecting csPCa (8, 10–15, 24).

Previous studies have revealed that patients with multi-lesions are not associated with higher risk of detecting csPCa (OR 1.05, 95% CI 0.60-1.84, p = 0.857), especially with PI-RADS 3 or 4 lesions (25). In addition, Günzel et al. determined that the added value of reduced SB scheme is low for PCa diagnosis and risk evaluation (26). However, the study by Darren et al. considered that multi-lesion csPCa might occur preferentially on the side of the index tumor (8). And most of the studies related to ips-SB+TB mainly focused on the patient with single MRI lesion, suggesting that ips-SB+TB would be feasible substitute for SB+TB in most cases with single MRI lesion (8, 13–15). While, the feasibility of the ips-SB+TB for the diagnosis of the patients with multi-lesions is still lack of support. However, in our study, ips-SB+TB still demonstrated comparable csPCa detection than SB+TB, and patients with multiple lesions were more likely to have csPCa than patients with a single lesion, especially with PIRADS 5 as the index lesion. This is consistent with the findings of Ruan et al, biopsy for a non-index lesion in patients with multiple lesions provided limited additional pathological information (11).

According to a study conducted by Nakanishi et al., patients with contralateral csPCa, if they have any of the following three predictive factors: being 75 years or older, having a PI-RADS score of 4 or higher, or a PSAD of 0.3 or higher. Therefore, utilizing the SB+TB biopsy schemes may be the most suitable approach for these patients (27). Furthermore, Wang et al. considered prior biopsy history (OR: 3.148; p=0.021) and inadequate biopsy experience (OR: 0.701; p=0.032) as the potential factors resulting in 10 upgrading on con-SB+TB (15). While Phelps et al. found no predictors of csPCa detected by con-SB+TB but missed by ips-SB+TB (14). In our study, ips-SB+TB underestimated 9 patients with csPCa and missed 4 patients with csPCa. No potential predictors relevant to csPCa were found. However, there is an overall lack of studies identifying risk factors associated with patients with csPCa contralateral to the index lesion and inconsistencies between SB+TB and surgical pathology findings. Therefore, further studies are needed to evaluate and identify standard factors.

Our study had several notable strengths. Firstly, all mpMRI scans were re-evaluated by both a radiologist and a urologist before biopsy, reducing discrepancies and inaccuracies in MRI interpretation. This also ensured the accuracy of target localization and cognitive-fusion TB. Thirdly, we included many patients with multi-lesions and showed that ips-SB+TB is still appropriate for these patients. However, there were some limitations to our study. Firstly, the study was conducted at a single center, limiting the generalizability of the results and requiring validation from other institutions. However, our main findings are broadly consistent with those of other centers (10, 12, 23, 28). Secondly, the use of the visual registration approach may have imposed some limitations on our findings. Nevertheless, previous research has shown that there is no significant difference in cancer detection between the cognitive-fusion and software-based methods when using the combined biopsy strategy (29, 30). Thirdly, as a retrospective study, it is subject to inherent biases. To address potential biases in retrospective study, we implemented strict patient selection criteria, multidisciplinary collaboration for MRI evaluation and biopsy planning, detailed pathological assessment, and comprehensive data collection and analysis methods. This approach aimed to enhance the internal validity and reliability of our research findings. Fourthly, our definition of csPCa (ISUP ≥2) does not incorporate risk-stratification criteria. This may reduce applicability in settings where treatment decisions rely on composite risk models. Future multi-center studies with larger surgical validation cohorts are warranted to confirm these findings.

## 5 Conclusions

In conclusion, our study demonstrated that ips-SB+TB showed comparable ability in detecting csPCa than SB+TB. The results of this study provide valuable insights into the potential of ips-SB+TB as an alternative to SB+TB, but further research is needed to optimize the diagnosis of prostate cancer and minimize the risks of underdiagnosis and overtreatment.

## Data Availability

The original contributions presented in the study are included in the article/**Supplementary Material**. Further inquiries can be directed to the corresponding authors.

## References

[1] Siegel RL Miller KD Fuchs HE Jemal A. Cancer statistics, 2022. CA: Cancer J Clin. (2022) 72:7–33. doi: 10.3322/caac.21708, PMID: 35020204

[2] Günzel K Cash H Buckendahl J Königbauer M Asbach P Haas M. The addition of a sagittal image fusion improves the prostate cancer detection in a sensor-based MRI/ultrasound fusion guided targeted biopsy. BMC urology. (2017) 17(1):7. doi: 10.1186/s12894-016-0196-9, PMID: 28086856 PMC5234255

[3] Ahdoot M Wilbur AR Reese SE Lebastchi AH Mehralivand S Gomella PT. MRI-targeted, systematic, and combined biopsy for prostate cancer diagnosis. New Engl J Med. (2020) 382:917–28. doi: 10.1056/NEJMoa1910038, PMID: 32130814 PMC7323919

[4] Kasivisvanathan V Rannikko AS Borghi M Panebianco V Mynderse LA Vaarala MH. MRI-targeted or standard biopsy for prostate-cancer diagnosis. New Engl J Med. (2018) 378:1767–77. doi: 10.1056/NEJMoa1801993, PMID: 29552975 PMC9084630

[5] Rouvière O Puech P Renard-Penna R Claudon M Roy C Mège-Lechevallier F. Use of prostate systematic and targeted biopsy on the basis of multiparametric MRI in biopsy-naive patients (MRI-FIRST): a prospective, multicentre, paired diagnostic study. Lancet Oncol. (2019) 20:100–9. doi: 10.1016/S1470-2045(18)30569-2, PMID: 30470502

[6] Drost FH Osses D Nieboer D Bangma CH Steyerberg EW Roobol MJ. Prostate magnetic resonance imaging, with or without magnetic resonance imaging-targeted biopsy, and systematic biopsy for detecting prostate cancer: A cochrane systematic review and meta-analysis. Eur urology. (2020) 77:78–94. doi: 10.1016/j.eururo.2019.06.023, PMID: 31326219

[7] van der Leest M Cornel E Israël B Hendriks R Padhani AR Hoogenboom M. Head-to-head comparison of transrectal ultrasound-guided prostate biopsy versus multiparametric prostate resonance imaging with subsequent magnetic resonance-guided biopsy in biopsy-naïve men with elevated prostate-specific antigen: A large prospective multicenter clinical study. Eur urology. (2019) 75:570–8. doi: 10.1016/j.eururo.2018.11.023, PMID: 30477981

[8] Bryk DJ Llukani E Taneja SS Rosenkrantz AB Huang WC Lepor H. The role of ipsilateral and contralateral transrectal ultrasound-guided systematic prostate biopsy in men with unilateral magnetic resonance imaging lesion undergoing magnetic resonance imaging-ultrasound fusion-targeted prostate biopsy. Urology. (2017) 102:178–82. doi: 10.1016/j.urology.2016.11.017, PMID: 27871829

[9] Siddiqui MM Rais-Bahrami S Turkbey B George AK Rothwax J Shakir N. Comparison of MR/ultrasound fusion-guided biopsy with ultrasound-guided biopsy for the diagnosis of prostate cancer. Jama. (2015) 313:390–7. doi: 10.1001/jama.2014.17942, PMID: 25626035 PMC4572575

[10] Diamand R Peltier A Roche JB Lievore E Lacetera V Chiacchio G. Optimizing multiparametric magnetic resonance imaging-targeted biopsy and prostate cancer grading accuracy. World J urology. (2023) 41:77–84. doi: 10.1007/s00345-022-04244-4, PMID: 36509932

[11] Ruan M Wang H Li X Song G. Novel sampling scheme with reduced cores in men with multiparametric MRI-visible lesions undergoing prostate biopsy. Abdominal Radiol (New York). (2023) 48:2139–47. doi: 10.1007/s00261-023-03894-1, PMID: 37036488

[12] Yusim I Mazor E Frumkin E Jabareen M Hefer B Elsaraya N. Evaluation of the optimal strategy in men with a single unilateral suspicious lesion on MRI undergoing transperineal MRI/ultrasound fusion prostate biopsy. Prostate. (2023) 83:1255–62. doi: 10.1002/pros.24585, PMID: 37263774

[13] Freifeld Y Xi Y Passoni N Woldu S Hornberger B Goldberg K. Optimal sampling scheme in men with abnormal multiparametric MRI undergoing MRI-TRUS fusion prostate biopsy. Urologic Oncol. (2019) 37:57–62. doi: 10.1016/j.urolonc.2018.10.009, PMID: 30446460

[14] Phelps TE Yilmaz EC Harmon SA Belue MJ Shih JH Garcia C. Ipsilateral hemigland prostate biopsy may underestimate cancer burden in patients with unilateral mpMRI-visible lesions. Abdominal Radiol (New York). (2023) 48(3):1079–89. doi: 10.1007/s00261-022-03775-z, PMID: 36526922 PMC10765956

[15] Wang F Chen T Wang M Chen H Wang C Liu P. Clinically significant prostate cancer (csPCa) detection with various prostate sampling schemes based on different csPCa definitions. BMC urology. (2021) 21:183. doi: 10.1186/s12894-021-00949-7, PMID: 34949183 PMC8697444

[16] Hagens MJ Fernandez Salamanca M Padhani AR van Leeuwen PJ van der Poel HG Schoots IG. Diagnostic performance of a magnetic resonance imaging-directed targeted plus regional biopsy approach in prostate cancer diagnosis: A systematic review and meta-analysis. Eur Urol Open science. (2022) 40:95–103. doi: 10.1016/j.euros.2022.04.001, PMID: 35540708 PMC9079161

[17] Sanguedolce F Lauwers CNG Tedde A Basile G Chernysheva D Uleri A. Regional versus systematic biopsy in addition to targeted biopsy: results from a systematic review and meta-analysis. Eur Urol Oncol. (2025) 8(2):534-43. doi: 10.1016/j.euo.2024.10.006, PMID: 39455339

[18] Wu Q Tu X Jiang J Ye J Lin T Liu Z. Is ipsilateral systematic biopsy combined with targeted biopsy the optimal substitute for bilateral systematic biopsy combined with targeted biopsy: A systematic review and meta-analysis. Urologic Oncol. (2025) 43:307–17. doi: 10.1016/j.urolonc.2024.11.023, PMID: 39710538

[19] Wu Q Wang J Tu X Chen B Jiang J Ye J. Optimizing the strategies to perform prostate biopsy in MRI-positive patients: a systematic review and network meta-analysis. EClinicalMedicine. (2025) 82:103164. doi: 10.1016/j.eclinm.2025.103164, PMID: 40212047 PMC11982038

[20] Zhang CC Tu X Lin TH Cai DM Yang L Nie L. The role of prostate-specific antigen density and negative multiparametric magnetic resonance imaging in excluding prostate cancer for biopsy-naïve men: clinical outcomes from a high-volume center in China. Asian J andrology. (2022) 24:615–9. doi: 10.4103/aja202220, PMID: 35532555 PMC9809478

[21] Turkbey B Rosenkrantz AB Haider MA Padhani AR Villeirs G Macura KJ. Prostate imaging reporting and data system version 2.1: 2019 update of prostate imaging reporting and data system version 2. Eur Urol. (2019) 76:340–51. doi: 10.1016/j.eururo.2019.02.033, PMID: 30898406

[22] Epstein JI Egevad L Amin MB Delahunt B Srigley JR Humphrey PA. The 2014 international society of urological pathology (ISUP) consensus conference on gleason grading of prostatic carcinoma: definition of grading patterns and proposal for a new grading system. Am J Surg pathology. (2016) 40:244–52. doi: 10.1097/PAS.0000000000000530, PMID: 26492179

[23] Yu L-P Du Y-Q Sun Y-R Qin C-P Yang W-B Huang Z-X. Value of cognitive fusion targeted and standard systematic transrectal prostate biopsy for prostate cancer diagnosis. Asian J Andrology. (2024) 26:479–83. doi: 10.4103/aja202414, PMID: 38783630 PMC11449411

[24] Drost FH Osses DF Nieboer D Steyerberg EW Bangma CH Roobol MJ. Prostate MRI, with or without MRI-targeted biopsy, and systematic biopsy for detecting prostate cancer. Cochrane Database systematic Rev. (2019) 4:Cd012663. doi: 10.1002/14651858.CD012663.pub2, PMID: 31022301 PMC6483565

[25] Patel N Halpern JA Kasabwala K Cricco-Lizza E Herman M Margolis D. Multiple regions of interest on multiparametric magnetic resonance imaging are not associated with increased detection of clinically significant prostate cancer on fusion biopsy. J urology. (2018) 200:559–63. doi: 10.1016/j.juro.2018.03.002, PMID: 29518433

[26] Günzel K Magheli A Busch J Baco E Cash H Heinrich S. Evaluation of systematic prostate biopsies when performing transperineal MRI/TRUS fusion biopsy with needle tracking-what is the additional value? Int Urol Nephrol. (2022) 54(10):2477–83. doi: 10.1007/s11255-022-03309-y, PMID: 35877030

[27] Nakanishi Y Ito M Kataoka M Ikuta S Sakamoto K Takemura K. Who Can Avoid Biopsy of Magnetic Resonance Imaging-Negative Lobes without Compromising Significant Cancer Detection among Men with Unilateral Magnetic Resonance Imaging-Positive Lobes? Urologia internationalis. (2021) 105:386–93. doi: 10.1159/000511636, PMID: 33242853

[28] Bourgeno H-A Jabbour T Baudewyns A Lefebvre Y Ferriero M Simone G. The added value of side-specific systematic biopsy in patients diagnosed by magnetic resonance imaging–targeted prostate biopsy. Eur Urol Oncol. (2024) 7(6):1320-26. doi: 10.1016/S0302-2838(24)00648-1, PMID: 38272745

[29] Elkhoury FF Felker ER Kwan L Sisk AE Delfin M Natarajan S. Comparison of targeted vs systematic prostate biopsy in men who are biopsy naive: the prospective assessment of image registration in the diagnosis of prostate cancer (PAIREDCAP) study. JAMA surgery. (2019) 154:811–8. doi: 10.1001/jamasurg.2019.1734, PMID: 31188412 PMC6563598

[30] Hamid S Donaldson IA Hu Y Rodell R Villarini B Bonmati E. The smartTarget biopsy trial: A prospective, within-person randomised, blinded trial comparing the accuracy of visual-registration and magnetic resonance imaging/ultrasound image-fusion targeted biopsies for prostate cancer risk stratification. Eur urology. (2019) 75:733–40. doi: 10.1016/j.eururo.2018.08.007, PMID: 30527787 PMC6469539

