# Supplementary material for: Assessing the diagnostic accuracy of unilateral systematic biopsy combined with targeted biopsy
Source: Front Oncol. 2025 Aug 19;15:1599869. doi: 10.3389/fonc.2025.1599869 (PMC12401680; doi:10.3389/fonc.2025.1599869)

Supplementary Material

Supplementary Table 1. Characters of patient with clinically significant prostate cancer missed by ips-SB+TB

| Patient number | Biopsy pathology for ips-SB+TB^*^ | Biopsy pathology for SB+TB^*^ | Age | PI-RADS Score for index lesion | Index lesion size (cm) | Lesion number | Location of the index lesion | tPSA (ng/mL) | fPSA (ng/mL) | PSAD | Prostate volume (ml) |
| --- | --- | --- | --- | --- | --- | --- | --- | --- | --- | --- | --- |
| 1 | 3+3 | 3+4 | 77 | 3 | 1.3 | 2 | ​​Right basal transitional zone | 11.7 | 2.93 | 0.395904 | 29.55264 |
| 2 | 3+3 | 3+4 | 68 | 5 | 1.5 | 2 | Left mid peripheral zone | 13.7 | 1.46 | 0.469127 | 29.2032 |
| 3 | 0 | 4+3 | 76 | 4 | 0.6 | 2 | Left apical peripheral zone | 11.9 | 3.65 | 0.187924 | 63.32352 |
| 4 | 3+3 | 4+4 | 80 | 4 | 1.2 | 2 | Lesions located in the left basal Lesions located in the left basal transition zone and left basal peripheral zone | 6.8 | 0.709 | 0.287564 | 23.64692 |

* Gleason Score.

Supplementary Table 2. csPCa positive cores rates of different biopsy schemes

| Biopsy sampling strategies | Total cores | csPCa Positive cores rate % (n) | P value (Compared with SB+TB) |
| --- | --- | --- | --- |
| SB+TB | 3744 | 15.0 (563) | - |
| TB | 744 | 29.7 (221) | < 0.001 |
| ips-SB+TB | 2244 | 21.0 (472) | < 0.001 |
| con-SB+TB | 2244 | 13.9 (312) | 0.2293 |

Supplementary Table 3. Kappa Statistics for Pathology Consistency among Different Biopsy Schemes

| Biopsy sampling strategies | Kappa | 95% CI | p value |
| --- | --- | --- | --- |
| All patients |  |  |  |
| ips-SB+TB compared with SB+TB | 0.89 | 0.85-0.92 | 0.00 |
| con-SB+TB compared with SB+TB | 0.85 | 0.82-0.89 | 0.00 |
| TB compared with SB+TB | 0.70 | 0.66-0.74 | 0.00 |
| PIRADS 3 |  |  |  |
| ips-SB+TB compared with SB+TB | 0.87 | 0.83-0.91 | 0.00 |
| con-SB+TB compared with SB+TB | 0.88 | 0.84-0.91 | 0.00 |
| TB compared with SB+TB | 0.68 | 0.63-0.72 | 0.00 |
| PIRADS 4 |  |  |  |
| ips-SB+TB compared with SB+TB | 0.88 | 0.85-0.91 | 0.00 |
| con-SB+TB compared with SB+TB | 0.87 | 0.84-0.90 | 0.00 |
| TB compared with SB+TB | 0.70 | 0.67-0.73 | 0.00 |
| PIRADS 5 |  |  |  |
| ips-SB+TB compared with SB+TB | 0.89 | 0.85-0.92 | 0.00 |
| con-SB+TB compared with SB+TB | 0.76 | 0.72-0.80 | 0.00 |
| TB compared with SB+TB | 0.61 | 0.56-0.66 | 0.00 |

Supplementary Table 4. Subgroup analysis of detection rate of PCa

| Biopsy strategies | PCa Detection rate  n (%) | P value | csPCa Detection rate  n (%) | P value |
| --- | --- | --- | --- | --- |
| PIRADS |  |  |  |  |
| PIRADS 3 (n=76) |  |  |  |  |
| SB+TB | 14 (18.4) | reference | 8 (10.5) | reference |
| TB | 10 (13.2) | 0.125 | 6 (7.9) | 0.5 |
| ips-SB+TB | 12 (15.8) | 0.5 | 7 (9.2) | 1 |
| con-SB+TB | 14 (18.4) | 1 | 7 (9.2) | 0.016 |
| PIRADS 4 (n=106) |  |  |  |  |
| SB+TB | 59 (55.7) | reference | 46 (43.4) | reference |
| TB | 48 (45.3) | <0.001 | 40 (37.7) | 0.031 |
| ips-SB+TB | 54 (50.9) | 0.0625 | 44 (41.5) | 0.5 |
| con-SB+TB | 56 (52.8) | 0.25 | 43 (40.6) | <0.001 |
| PIRADS 5 (n=68) |  |  |  |  |
| SB+TB | 53 (77.9) | reference | 49 (72.1) | reference |
| TB | 47 (69.1) | 0.031 | 45 (66.2) | 0.125 |
| ips-SB+TB | 50 (73.5) | 0.25 | 48 (70.6) | 1 |
| con-SB+TB | 51 (75.0) | 0.5 | 46 (67.6) | 0.625 |
| PSA (ng/ml) |  |  |  |  |
| PSA ≤ 10 (n=134) |  |  |  |  |
| SB+TB | 60 (44.8) | reference | 45 (33.6) | reference |
| TB | 49 (36.6) | <0.001 | 39 (29.1) | 0.031 |
| ips-SB+TB | 55 (41.0) | 0.0625 | 44 (32.8) | 1 |
| con-SB+TB | 56 (41.8) | 0.125 | 40 (29.9) | <0.001 |
| PSA > 10 (N=116) |  |  |  |  |
| SB+TB | 66 (56.9) | reference | 58 (50.0) | reference |
| TB | 56 (48.3) | 0.002 | 52 (50.0) | 0.031 |
| ips-SB+TB | 61 (52.6) | 0.063 | 55 (44.8) | 0.25 |
| con-SB+TB | 65 (56.0) | 1 | 56 (48.3) | 0.002 |
| PSAD (ng/ml/cc) |  |  |  |  |
| PSAD≤0.15 (n=90) |  |  |  |  |
| SB+TB | 21 (23.3) | reference | 11 (12.2) | reference |
| TB | 11 (12.2) | 0.002 | 10 (11.1) | 1 |
| ips-SB+TB | 16 (17.8) | 0.063 | 11 (12.2) | 1 |
| con-SB+TB | 18 (20.0) | 0.727 | 10 (11.1) | <0.001 |
| PSAD >0.15 (n=160) |  |  |  |  |
| SB+TB | 105 (65.6) | reference | 92 (57.5) | reference |
| TB | 94 (58.8) | <0.001 | 81 (50.6) | <0.001 |
| ips-SB+TB | 100 (62.5) | 0.063 | 88 (55) | 0.125 |
| con-SB+TB | 103 (64.4) | 0.5 | 86 (53.8) | <0.001 |
| Biopsy history |  |  |  |  |
| No prior biopsy (n=214) |  |  |  |  |
| SB+TB | 115 (53.7) | reference | 95 (44.4) | reference |
| TB | 98 (45.8) | <0.001 | 84 (39.3) | <0.001 |
| ips-SB+TB | 106 (49.5) | 0.004 | 92 (43.0) | 0.25 |
| con-SB+TB | 111 (51.9) | 0.125 | 88 (41.1) | <0.001 |
| With prior biopsy (n=36) |  |  |  |  |
| SB+TB | 11 (30.6) | reference | 8 (22.2) | reference |
| TB | 7 (19.4) | 0.125 | 7 (19.4) | 1 |

Supplementary Table 5. Univariable and multivariable logistic regression analysis for predictors of csPCa underestimated by ips-SB+TB

| Variables | Univariable | |  | Multivariable | |
| --- | --- | --- | --- | --- | --- |
|  | OR (95%CI) | p |  | OR (95%CI) | p |
| PIRADS |  |  |  |  |  |
| 3 | 1.00 (Reference) |  |  |  |  |
| 4 | 3.71 (0.42 ~ 32.44) | 0.236 |  |  |  |
| 5 | 2.27 (0.20 ~ 25.64) | 0.507 |  |  |  |
| Lesion number |  |  |  |  |  |
| Single lesion | 1.00 (Reference) |  |  | 1.00 (Reference) |  |
| Multiple lesions | 7.00 (0.85 ~ 57.76) | 0.071 |  | 5.36 (0.63 ~ 45.29) | 0.123 |
| Biopsy history |  |  |  |  |  |
| 1 | 1.00 (Reference) |  |  |  |  |
| 2 | 2.04 (0.40 ~ 10.52) | 0.395 |  |  |  |
| Prostate volume (ml) |  |  |  |  |  |
| ≤ 40 | 1.00 (Reference) |  |  | 1.00 (Reference) |  |
| > 40 | 0.18 (0.04 ~ 0.93) | 0.041 |  | 0.33 (0.03 ~ 4.19) | 0.392 |
| Lesion Size | 0.61 (0.18 ~ 2.01) | 0.415 |  |  |  |
| PSA | 0.99 (0.89 ~ 1.10) | 0.804 |  |  |  |
| fPSA | 1.00 (0.56 ~ 1.77) | 0.994 |  |  |  |
| PSAD | 2.84 (0.19 ~ 41.69) | 0.446 |  |  |  |

**Supplementary figure 1. The flow chart of the patient selection**

**
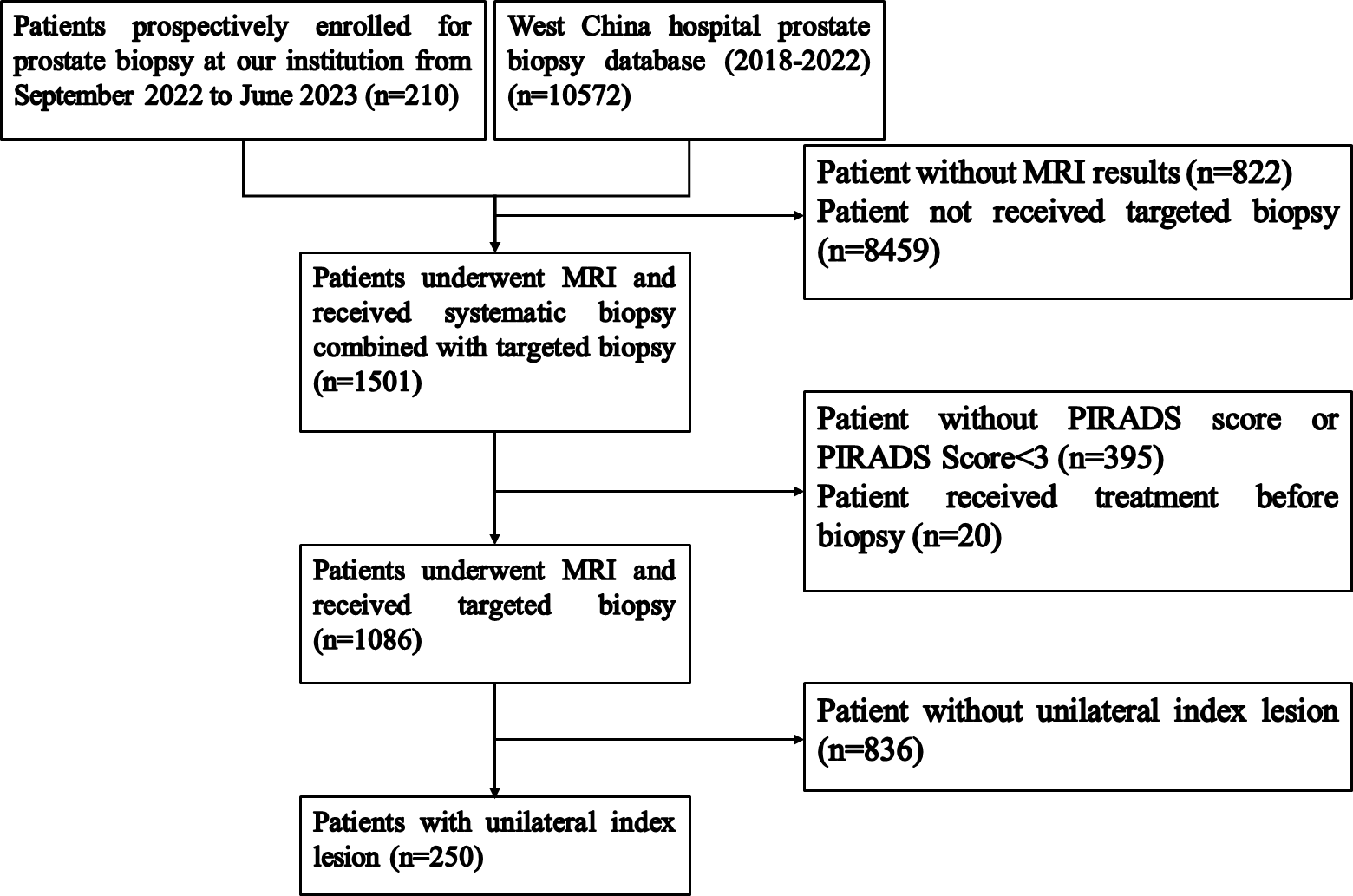
**

**Supplementary figure 2. Pattern diagram for Prostate biopsy.**


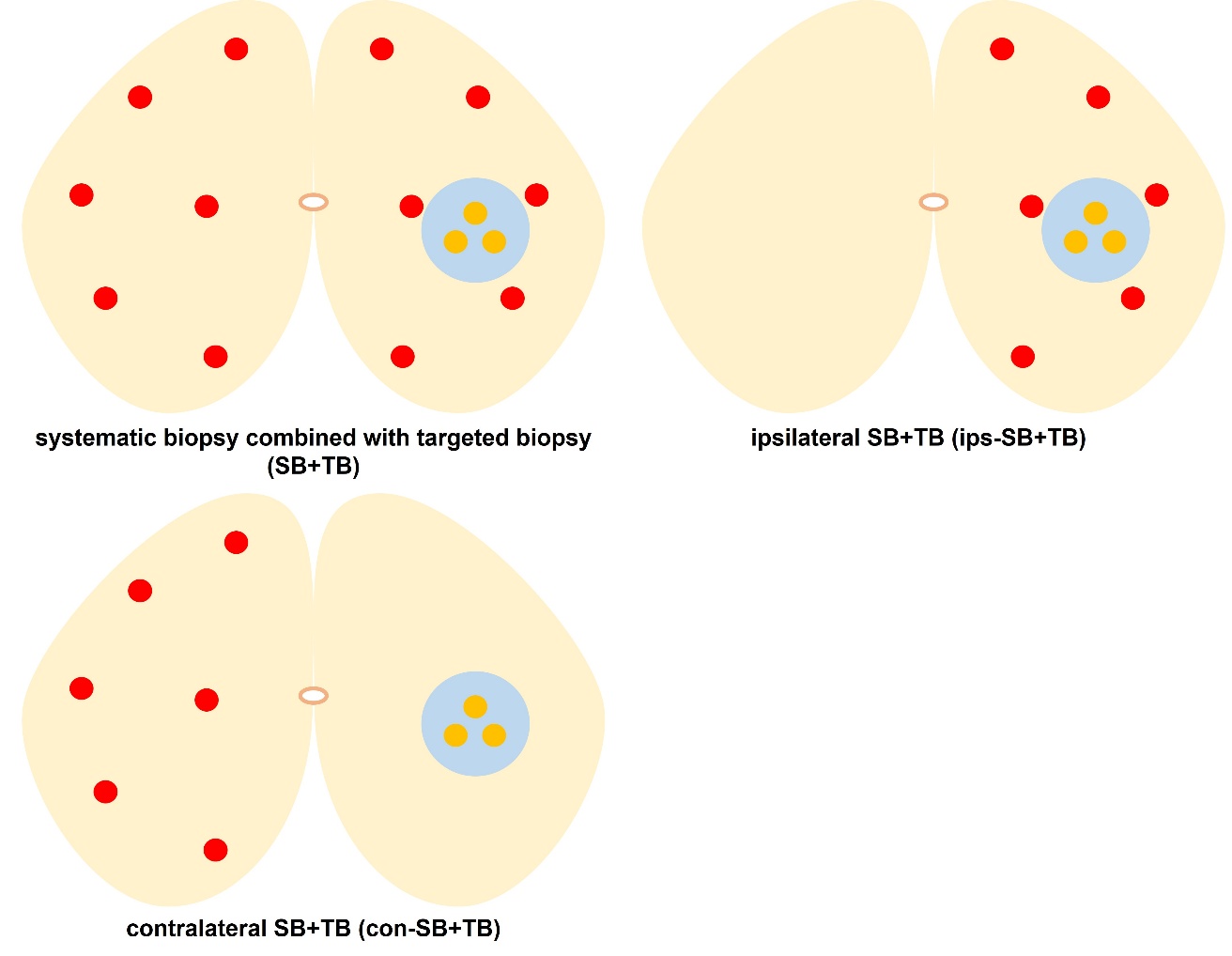


**Supplementary figure 3. The subgroup analysis of csPCa detection rate for ips-SB+TB lesion number on the detection rate based on lesion numbers**


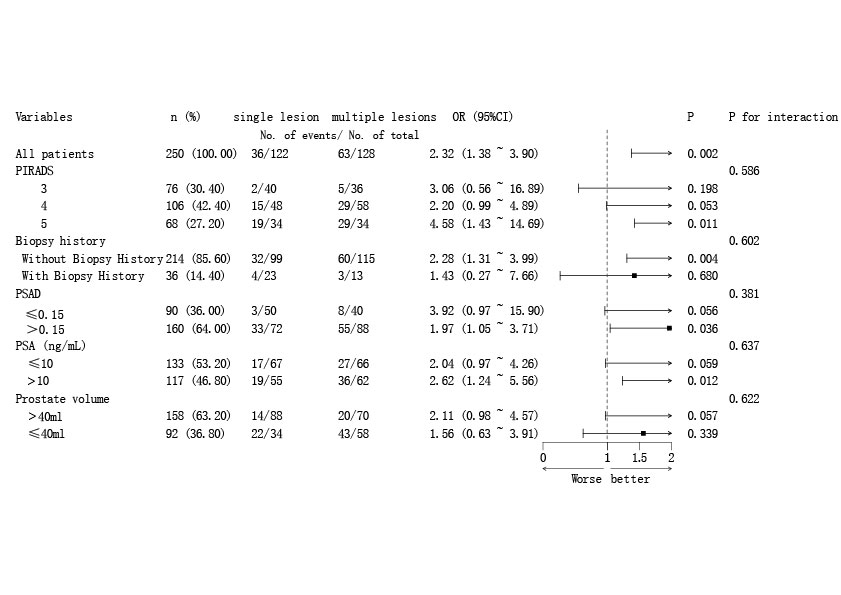

Supplement: Supplementary file 1 [file DataSheet1.docx]
